# Long COVID and Subjective Wellbeing among U.S. Working-Age Adults

**DOI:** 10.21203/rs.3.rs-9707617/v1

**Published:** 2026-06-08

**Authors:** Douglas A Wolf, Shannon M Monnat, Iliya Gutin, Emily W. Wiemers, Jennifer Karas Montez, Qiyu Deng

**Affiliations:** Syracuse University; Syracuse University; Syracuse University; Syracuse University; Syracuse University; Syracuse University

**Keywords:** COVID-19, long COVID, subjective wellbeing, National Wellbeing Survey

## Abstract

**Background:**

Long COVID symptoms can persist for months or years, impeding daily functioning, employment, and social relationships. While prior research links long COVID to adverse clinical mental health outcomes, less attention has been paid to broader subjective wellbeing—including life satisfaction, happiness, and hopefulness. This study examined associations between long COVID symptoms and subjective wellbeing among U.S. working-age adults, including whether associations differ by sex.

**Methods:**

This cross-sectional analysis used data from the 2023 and 2024 National Wellbeing Surveys (NWS), a weighted survey of U.S. adults aged 18–64 (N = 13,990). We operationalized long COVID as having experienced any of eight symptoms (fatigue, forgetfulness, shortness of breath, joint or muscle pain, rapid heartbeat, dizziness, depression or anxiety, and exercise-exacerbated symptoms), a symptom count, and a dose-response specification. Subjective wellbeing measures include life satisfaction, happiness, and hopefulness. We estimated regression models for the full sample and separately by sex, adjusting for sociodemographic and economic characteristics. We reported results using point estimates, odds ratios (ORs) and 95% Confidence Intervals (95% CI).

**Results:**

Nearly 39% (95% CI: 37.5,40.2) of respondents reported at least one long COVID symptom, with females reporting higher prevalence (43.1%) than males (34.7%) and more symptoms (mean = 1.80) than males (mean = 1.35). Long COVID was significantly associated with lower wellbeing across all three subjective wellbeing outcomes for both sexes. A dose-response pattern was observed: more symptoms were associated with progressively worse wellbeing. Among individual symptoms, depression or anxiety and cognitive difficulties exhibited the strongest negative associations with subjective wellbeing. Despite reporting higher long COVID prevalence, females had higher predicted life satisfaction and hopefulness than males at most levels of long COVID severity.

**Conclusions:**

In this nationally representative sample of U.S. working-age adults, long COVID was associated with reduced subjective wellbeing across multiple operationalizations of long COVID and dimensions of wellbeing for both males and females. These findings underscore that the burden of long COVID extends beyond clinical mental health outcomes to broader evaluative and experiential dimensions of wellbeing, suggesting long COVID may represent an important population health risk for diminished quality of life.

## Introduction

The post-acute sequelae of SARS-CoV-2 infection, or long COVID, continues to be a major public health concern, affecting the health and economic wellbeing of millions of Americans. Long COVID symptoms span multiple domains, including fatigue, cognitive impairment, shortness of breath, joint and muscle pain, heart palpitations, and dizziness [1], and frequently persist for months or years, impeding daily functioning, employment, social relationships, and overall quality of life [2, 3].

Estimates of long COVID prevalence in the United States vary widely depending on data source and measurement strategy, but U.S. population-based studies suggest that a nontrivial share of adults experience symptoms lasting three months or longer, with estimates ranging from 9% to 63% among those with prior COVID-19 infection [4–8]. This variation reflects differences in long COVID definitions, symptom inventories, study populations, and timing of survey administration [9]. Nevertheless, the range underscores the substantial and ongoing public health burden of long COVID in the U.S. population.

A growing body of research demonstrates that long COVID is associated with adverse mental health outcomes. Individuals with long COVID report higher levels of anxiety, depression, and posttraumatic stress symptoms compared to those without long COVID [2, 10–14]. In addition to clinical mental health outcomes, one community-based study in Texas found that long COVID is associated with decreased life satisfaction [15], and a study based on a sample of adults in Victoria, Australia showed that long COVID was associated with an increase in negative emotions [16]. However, the literature remains heavily focused on clinical mental health indicators, with comparatively less attention to subjective wellbeing, especially in nationally representative samples. Subjective wellbeing encompasses broad aspects of how individuals experience and evaluate their lives, including life satisfaction, happiness, and hope for the future. Critically, these subjective dimensions of wellbeing may respond differently to long COVID than traditional clinical mental health outcomes. Understanding the relationship between long COVID and subjective wellbeing is important because subjective wellbeing is not only a key indicator of quality of life but is also linked to physical health and longevity [17, 18].]

There are multiple plausible pathways through which long COVID may diminish subjective wellbeing. Physiological processes associated with long COVID, including inflammation, autonomic dysfunction, and neurological changes, may directly affect mood and cognition [13, 19, 20]. Symptoms such as chronic fatigue, difficulty thinking, concentrating, or remembering (“brain fog”), and shortness of breath can disrupt daily functioning and reduce individuals’ capacity to engage in valued activities, thereby diminishing wellbeing [21]. Long COVID is also associated with elevated rates of depression and anxiety [10, 12], which are themselves strong predictors of reduced subjective wellbeing [22]. Beyond the biological, long COVID exerts substantial economic consequences that are also likely to undermine wellbeing. Long COVID symptoms can impair work capacity, increase absenteeism, and reduce earnings, contributing to risk of job loss and financial strain [11, 23]. Among working-age adults in the United States, estimates of lost earnings attributable to long COVID reached approximately $218 billion in 2023 [11]. The inability to maintain employment, sustain income, or fulfill occupational roles can threaten purpose, identity, and meaning in life [24]. Social mechanisms are also likely important. Limitations in physical functioning and ongoing health concerns may restrict social participation, strain interpersonal relationships, and increase stigma, social isolation, and loneliness [3, 14].

Despite growing recognition of the long-term burdens of long COVID on population health, important gaps remain. First, many existing studies rely on clinical or geographically limited samples, limiting generalizability. Second, prior work has largely focused on binary indicators of long COVID or single-symptom measures, which may obscure heterogeneity in symptom burden and severity and the potentially dose-dependent nature of its wellbeing consequences. Third, most studies emphasize clinical mental health outcomes such as depression or anxiety, rather than broader measures of subjective wellbeing that capture how individuals evaluate and experience their lives. Finally, although studies consistently show higher prevalence of long COVID and greater symptom burden among females [5, 7], it is unknown whether the relationship between long COVID and subjective wellbeing differs by sex.

The present study addresses these gaps using national data on adults aged 18–64 in the United States collected in 2023 and 2024. We examine associations between self-reported symptoms of long COVID and three distinct dimensions of subjective wellbeing—life satisfaction (evaluative wellbeing), happiness (hedonic wellbeing), and hopefulness (a forward-looking aspirational measure of wellbeing)—among males and females. We consider multiple operationalizations of long COVID: any experience of long COVID symptoms, a total count of eight symptoms experienced, and a dose-response specification. We also investigate differences among the eight potential symptoms in their contributions to wellbeing. By moving beyond clinical mental health indicators to examine subjective wellbeing more broadly, and by employing a nationally representative sample of adults ages 18–64 alongside a rigorous long-COVID multi-operationalization approach, this study makes a novel contribution to the growing literature on the long-term consequences of COVID-19 for U.S. population wellbeing.

## Methods

### Study Population

This study uses data from the 2023 and 2024 National Wellbeing Surveys (NWS) [25, 26]. The NWS is cross-sectional survey designed to collect data on the health and wellbeing of adults ages 18–64 in the United States. The survey is administered by Qualtrics panels to a non-probability sample of adults who can read English. The NWS incorporates survey weights to account for sampling design and to improve representativeness of the U.S. adult population. Details regarding the NWS sampling design, recruitment procedures, survey administration, response rate, and representativeness are described in the NWS Methodology Report [27]. We used the 2023 and 2024 NWS since those are the only two years that included questions about long COVID symptoms. Institutional Review Board approval for the study was not required because we do not have access to identifying information.

We restricted the analytic sample to respondents with complete information on COVID and long COVID status, subjective wellbeing measures, and key covariates. From a potential sample of 13,990 respondents, 91 (about 0.7%) were lost due to missingness on one or more survey items, producing a final analysis sample of 13,899.

### COVID infection and long COVID

NWS respondents were asked whether they had ever experienced each of the following: they tested positive for coronavirus; suspected they had coronavirus based on symptoms but did not take a test to confirm; suspected they had coronavirus based on symptoms but tested negative; or received medical care or were hospitalized because of coronavirus. The implied time frame to which these questions pertain is the period since the onset of COVID-19 in early 2020. We coded those responding “yes” to any these items as having had prior COVID infection.

To identify individuals who experienced long COVID symptoms, NWS respondents in the “prior COVID infection” category were asked “Did you have any of the symptoms or health effects listed below that lasted for 3 months or longer that you did not have prior to having coronavirus?”: tiredness or fatigue; difficulty thinking or concentrating or forgetfulness/memory problems (sometimes referred to as “brain fog”); difficult breathing or shortness of breath; joint or muscle pain; fast-beating or pounding heart (also known as heart palpitations) or chest pain; dizziness on standing; depression, anxiety, or mood changes; and symptoms that get worse after physical or mental activities. These eight symptoms are specifically enumerated as part of the long COVID case definition [1, 28]. We coded (1) a binary variable indicating whether any such symptoms were reported (“any long COVID”), (2) the count of reported symptoms, ranging from 0 to 8 (“number of reported symptoms”), and (3) binary variables for the presence of each of the eight symptoms (using the 8 symptom labels listed above). The survey did not specifically ask respondents to self-diagnose as long COVID patients.

### Subjective wellbeing measures

We use a life satisfaction scale, comprising five items [29]. Respondents were asked, “Now please think about your life as a whole. Please indicate how much you agree or disagree with the following statements”: (1) In most ways, my life is ideal; (2) The conditions of my life are excellent; (3) I am satisfied with my life; (4) So far, I have gotten the important things I want in life; and (5) If I could live my life over, I would change almost nothing. Response options ranged from 1 = strongly disagree to 5 = strongly agree, resulting in summed scores that ranged from 5 to 25. We rescaled this summed life satisfaction score to lie on the 0–1 interval. Our indicator of happiness is based on the question: “Taking all things together, would you say you are: very happy, rather happy, not very happy, or not at all happy”? We dichotomized participants into “rather” or “very” happy = 1 and “not very happy” or “not at all happy”=0. Our indicator of hopefulness is based on the question that used a 5-category Likert scale ranging from Strongly disagree to Strongly agree: “Now please think about your life as a whole. Please indicate how much you agree or disagree with the following statement: “I am hopeful for the future”. We dichotomized participants into “somewhat” or “strongly” agree = 1 and “neither agree or disagree”, “somewhat disagree”, or “strongly disagree”=0.

### Other study measures

Our multivariable analyses control for several potential confounders shown in past research to be associated with the probability of experiencing long COVID. These include sex, race, education, employment status, household composition, marital status, political leanings (measured by vote cast in the 2020 Presidential election), and county metropolitan status. Females are more likely to develop long COVID and experience greater symptom burden than males [11, 30–32]. Several studies have also found differences in long COVID prevalence by race [11, 33, 34], educational attainment [11, 30, 32, 33], employment status [11, 23, 35], and the presence of children in the household [11, 36]. Divorced or separated adults face higher long COVID risk compared to married adults [11]. Political partisanship has been shown to be associated with COVID-19-related health behaviors and mortality [37, 38], and nonmetropolitan residents have higher long COVID prevalence than metropolitan residents, a difference that falls within a broader context of higher rural COVID-19 burden [39–43]. We also control for survey year, as long COVID prevalence in the United States has shifted over time, potentially due to changes in variant circulation, vaccination coverage, and population immunity [44] The categories for all covariates are shown in Table 1.

### Statistical analysis

We present results from both descriptive analysis and multivariable regression analyses, conducted with the entire analytic sample and separately by sex. Descriptive analyses include reporting the prevalence of any long COVID symptoms, average number of symptoms, and the prevalence of each symptom, along with averages of control variables. We used multivariate ordinary least-squares regressions to estimate associations between long COVID and life satisfaction and multivariate logistic regressions for the binary outcomes “happy” and “hopeful”.

For each outcome we considered four specifications: specification (1) includes the “any long COVID” binary variable; specification (2) estimates the relationship as linear in the number of symptoms (using the “number of reported symptoms” variable); specification (3) relaxes the linearity assumption, using binary indicators for having one, two, and up to eight long COVID symptoms; and specification (4)—of which there are 8 variants—includes a binary indicator for one of the 8 symptoms and adds as a control the count of additional reported symptoms. In all four specifications we control for the confounders described above. All analyses use survey weights supplied with the NWS data.

## Results

### Prevalence of Long COVID and Subjective Wellbeing

Summary statistics for the full sample, as well as separately by sex, are presented in Table 1. Just over 59% of the sample reported any prior COVID-19 infection. The prevalence of long COVID (i.e., those reporting any long COVID symptoms) in the full sample is nearly 39% (95% CI: 37.5, 40.2). This estimate of the prevalence of long COVID includes in its denominator the nearly 41% of the sample coded as not having had a previous COVID infection, and consequently not asked the survey questions about long COVID symptoms. When we condition on reported prior experience, our estimate of long COVID prevalence among the previously exposed population rises to 65%, Symptoms range in prevalence from 13.6% (12.6, 14.5) for heart palpitations or chest pains to 27.5% (26.2, 28.7) for tiredness or fatigue. The average life satisfaction score is 0.55 (0.545, 0.558) (on a range of 0 to 1). Nearly 84% (82.8, 84.7) of the sample reported being happy, and 72.1% (70.9, 73.3) reported being hopeful for the future.

Regarding sex differences, females reported substantially higher long COVID prevalence, 43.1% (41.2, 45.0), than males, 34.7% (32.8, 36.6). Females also reported a greater mean number of long COVID symptoms than males (1.798; [1.704, 1.892] vs. 1.35 [1.264, 1.437]). Females were also more likely than males to report each of the eight long COVID symptoms, with the largest absolute differences observed for difficulty thinking (25.4% [23.8, 27.0]) vs. 16.3% [14.8, 17.8]), tiredness or fatigue (31.7% [29.9, 33.5] vs. 23.4% [21.8, 25.1]), and depression or anxiety (24.7% [23.1, 26.3] vs. 18.8% [17.2, 20.3]). Despite these differences in long COVID burden, females were significantly more likely to report being hopeful for the future, 75.2% (73.6, 76.7) vs. 69.2% (67.3,71.0). Life satisfaction and happiness did not differ by sex.

### Associations between Long COVID and Subjective Wellbeing

Results from multivariable analyses are reported in Tables 2 (for the full sample), 3 (males) and 4 (females). In models estimating the overall association between any long COVID symptoms and subjective wellbeing while adjusting for covariates ([1], Table 2), long COVID was negatively associated with all three wellbeing outcomes. Reporting any long COVID symptoms was associated with a 0.063-point decrease (95% CI:−0.08, −0.045) on the life satisfaction scale and with lower odds of reporting happiness (OR = 0.606, 95% CI: 0.494, 0.743) and hopefulness (OR = 0.744, 95% CI: 0.626, 0.844), net of prior COVID infection and sociodemographic covariates. Having had any COVID infection without long COVID symptoms was not associated with any wellbeing outcome.

When the number of long COVID symptoms was entered as a continuous measure ([2], Table 2), each additional symptom was associated with a 0.014-point decrease in life satisfaction (95% CI:−0.017, −0.011) and with lower odds of happiness (OR = 0.878, 95% CI:0.850, 0.906) and hopefulness (OR = 0.938; 95% CI:0.921, 0.965). This pattern of associations between outcomes and the count of long COVID symptoms was similar between males and females in the sex-stratified models ([2], Tables 3 and 4).

**Figure 1:**
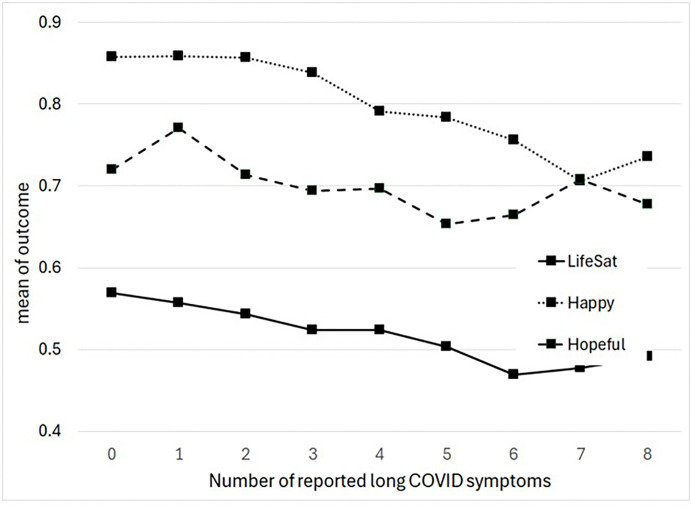
Levels of wellbeing by number of reported long COVID symptoms, full sample

When we relaxed the linearity assumption by including indicators for each symptom count from 1 to 8 ([3], Table 2), a non-linear dose-response pattern emerged. Participants reporting only one or two long COVID symptoms generally did not differ from those with no symptoms on any wellbeing outcome. Associations became progressively larger at higher symptom counts. For life satisfaction, coefficients ranged from − 0.023 for 1 symptom (95% CI: −0.049, −0.003) to − 0.112 for 6 symptoms, (95% CI: −0.145, −0.078). For happiness, the largest negative associations were observed among those reporting 7 symptoms (OR = 0.244; 95% CI: 0.142, 0.418) and 8 symptoms (OR = 0.412; 95% CI: 0.305, 0.558). For hopefulness, respondents with 5 symptoms (OR = 0.591; 95% CI: 0.436, 0.802) and 6 symptoms (OR = 0.622; 95% CI: 0.437, 0.886) showed the most pronounced reductions. Notably, respondents reporting all 8 symptoms showed somewhat attenuated associations compared to those reporting fewer symptoms, suggesting a slight nonlinearity at the highest severity levels. Predicted wellbeing values across symptom counts are shown in Fig. 1.

**Figure 2:**
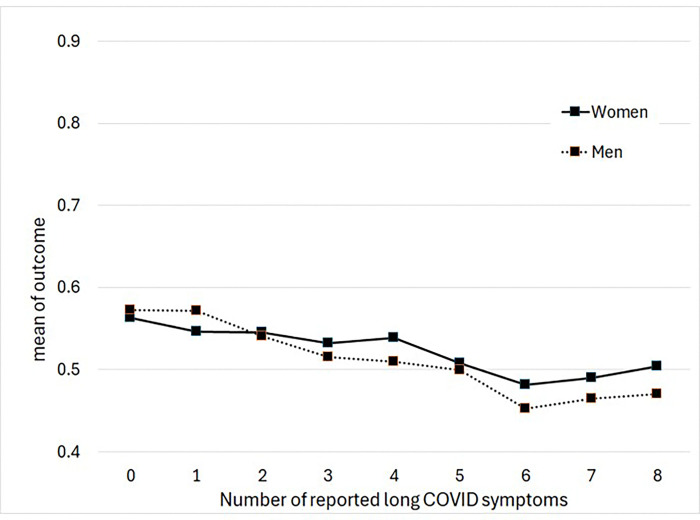
Levels of life satisfaction by number of reported long COVID symptoms, by sex

**Figure 3:**
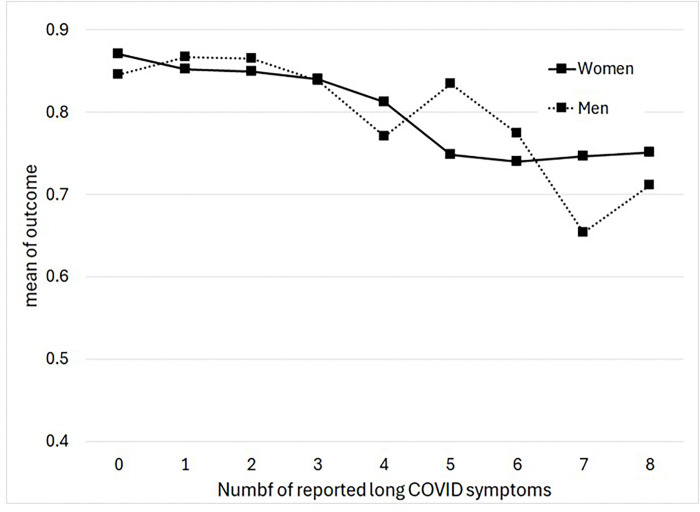
Levels of happiness by number of reported long COVID symptoms, by sex

**Figure 4:**
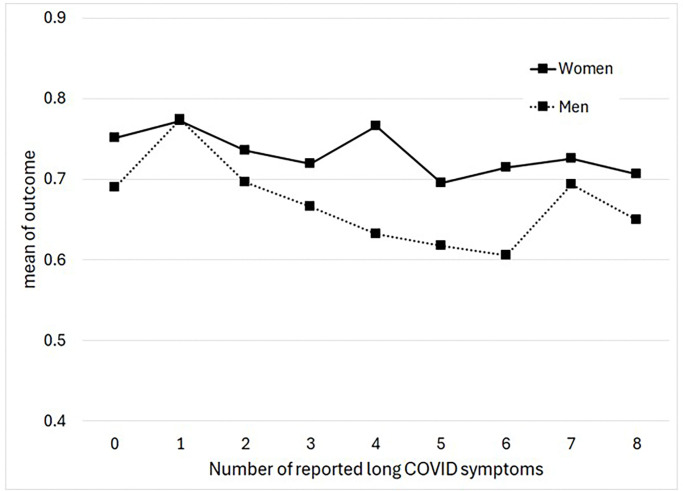
Levels of hopefulness by number of reported long COVID symptoms, by sex

Sex-stratified dose-response models (Tables 3 and 4) revealed broadly similar patterns for males and females. For both sexes, the strongest negative associations with life satisfaction were observed at 6 and 7 symptoms. Predicted wellbeing was nearly always higher for females than for males at a given symptom count for life satisfaction and hopefulness (Figs. 2 and 4). No consistent sex pattern was observed for happiness, though males showed a steeper dose-response gradient (Fig. 3).

Finally, models examining the independent contribution of each symptom to subjective wellbeing, while controlling for the count of other symptoms (specification [4], Table 2) revealed substantial heterogeneity across symptom types. Two symptoms related to mental health and cognition—depression or anxiety and difficulty thinking—exhibited the strongest and most consistent negative associations with subjective wellbeing. Depression or anxiety was associated with a 0.071-point reduction in life satisfaction (95% CI: −0.089, −0.002) and considerably lower odds of happiness (OR = 0.515; 95% CI: 0.402, 0.660) and hopefulness (OR = 0.702; 95% CI: 0.576, 0.856). Difficulty thinking (i.e., brain fog) was associated with lower life satisfaction (β=−0.024; 95% CI: −0.045, −0.004), happiness (OR = 0.576; 95% CI: 0.433, 0.767), and hopefulness (OR = 0.7; 95% CI: 0.560, 0.874). These patterns held in sex-stratified analyses: among males, depression or anxiety was associated with a 0.096-point decrease in life satisfaction (95% CI: −0.124, −0.069) and substantially lower odds of happiness (OR = 0.367; 95% CI: 0.255, 0.526). Among females, difficulty thinking was associated with lower happiness (OR = 0.550; 95% CI: 0.381, 0.795) and hopefulness (OR = 0.607; 95% CI: 0.461, 0.799). The remaining physical symptoms (fatigue, shortness of breath, joint or muscle pain, heart palpitations, dizziness on standing, and post-exertional symptom worsening) were generally not independently associated with subjective wellbeing after accounting for total symptom count.

## Discussion

Using data from the 2023 and 2024 National Wellbeing Surveys, this study examined associations between long COVID symptoms and three dimensions of subjective wellbeing among U.S. adults ages 18–64. Our findings demonstrate that long COVID is significantly and negatively associated with life satisfaction, happiness, and hopefulness across multiple operationalizations of long COVID symptom burden. A dose-response pattern was evident: wellbeing declined progressively as the number of reported long COVID symptoms increased, though respondents reporting only one or two symptoms generally did not differ from those without symptoms. Among individual symptoms, depression or anxiety and cognitive difficulty (i.e., brain fog) exhibited the strongest and most consistent negative associations with subjective wellbeing, while physical symptoms such as fatigue, shortness of breath, and joint pain were not independently associated with subjective wellbeing after accounting for total symptom burden. Associations were observed for both males and females, and findings from sex-stratified models were broadly consistent with the pooled results.

These findings extend prior research linking long COVID to adverse mental health outcomes such as clinical depression and anxiety [11, 12] by demonstrating that long COVID is also associated with diminished subjective wellbeing—a construct that captures how individuals evaluate and experience their lives beyond the presence or absence of diagnosable mental health conditions. While one community-based study found that long COVID was associated with decreased life satisfaction [15], our study builds on that study by using a nationally representative sample of working-age adults, by examining multiple dimensions of wellbeing simultaneously, and by demonstrating that these associations follow a dose-response pattern across symptom burden.

The finding that depression or anxiety and brain fog were the symptoms most strongly associated with reduced subjective wellbeing merits particular attention. Although conceptual proximity between these symptoms and subjective wellbeing outcomes might partially account for this pattern, these results also align with evidence that the psychological sequelae of long COVID, including depression and anxiety, function as pathways through which long COVID undermines broader wellbeing [10, 22]. Long COVID-related depression and brain fog may impair individuals' capacity to engage in valued activities, sustain social relationships, and maintain a sense of purpose and meaning in life [21, 24]. That physical symptoms were not independently associated with wellbeing after adjusting for symptom count suggests that the psychological and cognitive sequelae of long COVID may be more consequential for subjective wellbeing than physical symptoms alone, and that addressing these dimensions should be a priority in post-COVID care.

The dose-response relationship between symptom count and wellbeing is also noteworthy. The finding that one or two symptoms were generally insufficient to reduce wellbeing, while higher symptom counts were associated with progressively larger hits to wellbeing, suggests a threshold or cumulative burden dynamic. This has implications for clinical screening and intervention. Individuals reporting multiple persistent symptoms may warrant targeted monitoring not only for physical health but also for deterioration in broader psychosocial wellbeing. The slight attenuation of associations at the very highest symptom count (8 of 8) may reflect adaptation, selection, or differential help-seeking among the most severely affected individuals, though this pattern should be interpreted cautiously.

Our estimate of the prevalence of long COVID among those previously exposed—65%—is at the upper end of the large range of comparably defined estimates found in the literature. Our estimate likely reflects the NWS measurement approach: respondents were asked about eight specific symptoms lasting three months or longer without being asked to self-identify as having long COVID, which may capture a broader range of individuals than studies requiring explicit self-diagnosis or clinical confirmation. Additionally, the NWS assesses cumulative symptom experience rather than current symptoms, which may capture individuals whose symptoms have since resolved. Our estimate, especially in the context of substantial uncertainty regarding the prevalence of long COVID, suggests that the true incidence of long COVID is much higher than its diagnosed incidence, and that physicians should monitor the presence of relevant symptoms.

We acknowledge several limitations of the data and analysis. First, as a cross-sectional study, we cannot establish causal relationships between long COVID symptoms and subjective wellbeing. It is possible that individuals with lower baseline wellbeing are more likely to report persistent symptoms. Second, long COVID status was based on self-reported symptoms rather than clinical diagnosis, which may introduce misclassification. The NWS did not ask respondents to self-identify as having long COVID but rather assessed whether they experienced specific symptoms for three or more months that they did not have prior to experiencing COVID infection. While this approach has limitations, it is consistent with the symptom-based operationalization used in other population-based surveys, including the NHIS and BRFSS [7]. Third, the NWS captures cumulative long COVID symptom experience rather than current symptom status, meaning that some respondents may no longer be experiencing symptoms at the time they reported on their wellbeing. This likely attenuates estimated associations, making our findings conservative.

Despite these limitations, this study makes several contributions to the literature on long COVID and population wellbeing. It is among the first to examine associations between long COVID and subjective wellbeing using a national sample of U.S. working-age adults. By employing multiple operationalizations of long COVID, including binary, continuous, dose-response, and symptom-specific specifications, it provides a more detailed picture of how long COVID symptom burden relates to subjective wellbeing than studies relying on a single binary indicator. By moving beyond clinical mental health outcomes to examine evaluative, hedonic, and aspirational dimensions of wellbeing, our findings indicate that the consequences of long COVID for how people experience and evaluate their lives extend well beyond what clinical indicators alone would reveal. Together, these findings suggest that long COVID represents a risk to population wellbeing among a considerable share of U.S. working-age adults, with implications for clinical care, workplace policy, and public health surveillance.

## Conclusions

In this national sample of nearly 14,000 U.S. adults ages 18–64, long COVID symptoms were significantly associated with lower levels of subjective wellbeing in a dose-dependent manner, with depression or anxiety and brain fog exhibiting the strongest independent associations. These patterns were consistent across multiple dimensions of wellbeing and for both males and females. The findings underscore that the burden of long COVID extends beyond clinical health outcomes to encompass how individuals evaluate and experience their lives, highlighting the need for integrated approaches to post-COVID care that address psychological and cognitive symptoms alongside physical health, and for continued public health surveillance of long COVID and subjective wellbeing among working-age adults.

## Supplementary Material

Supplementary Files

This is a list of supplementary files associated with this preprint. Click to download.

• Tables.docx

Tables

Tables 1 to 4 are available in the Supplementary Files section.

## Data Availability

The data used in this study are publicly available through ICPSR (https://www.icpsr.umich.edu/web/NAHDAP/series/2340).
